# Recent Advances in Phytohormone Regulation of Apple-Fruit Ripening

**DOI:** 10.3390/plants10102061

**Published:** 2021-09-30

**Authors:** Yinglin Ji, Aide Wang

**Affiliations:** Key Laboratory of Fruit Postharvest Biology (Liaoning Province), Key Laboratory of Protected Horticulture (Ministry of Education), National & Local Joint Engineering Research Center of Northern Horticultural Facilities Design & Application Technology (Liaoning), College of Horticulture, Shenyang Agricultural University, Shenyang 110866, China; jiyinglin3@163.com

**Keywords:** apple, fruit ripening, phytohormones, regulation

## Abstract

Apple (*Malus domestica*) is, globally, one of the largest fruits in terms of cultivated area and yield. Apple fruit is generally marketed after storage, which is of great significance for regulating the market supply in the off-season of fruit production. Apple-fruit ripening, which culminates in desirable changes in structural and textural properties, is governed by a complex regulatory network. Much is known about ethylene as one of the most important factors promoting apple-fruit ripening. However, the dynamic interplay between phytohormones also plays an important part in apple-fruit ripening. Here, we review and evaluate the complex regulatory network concerning the action of phytohormones during apple-fruit ripening. Interesting future research areas are discussed.

## 1. Introduction

The apple (*Malus domestica*) is a fruit with a good range of cultivars, which have different ripening rates; depending on the different ripening rates, it may be supplied for market year-round from harvest [1]. As a typical climacteric fruit, apples have a peak in respiration and a burst of ethylene to unleash the ripening process in an autocatalytic response just prior to the initiation of ripening [2]. Apple-fruit ripening is mainly regulated by the phytohormone ethylene [2]. Therefore, it appears to be possible to control the storage life of apple fruit by regulating ethylene biosynthesis. For example, treatment with the compound ethephon, which is converted into ethylene in planta, promotes ethylene production and apple-fruit ripening [3], while 1-methylcyclopropene (1-MCP, an ethylene antagonist) treatment significantly blocks ethylene production and apple-fruit ripening [4]. Knowledge on the role of phytohormones other than ethylene during apple-fruit ripening has been limited for a long time. However, an increasing number of studies point to important roles for jasmonates, auxins, brassinosteroids, and abscisic acid in apple-fruit ripening [4,5,6,7]. A better understanding of the hormone regulatory mechanisms in the ripening of apple-fruit is both biologically meaningful and economically significant for generating strategies to improve apple-fruit qualities and fruit nutrition, and reduce postharvest economic losses [8]. In this current review, we summarize recent research advances in the phytohormone regulation of apple-fruit ripening and discuss future perspectives in this field. 

## 2. Ethylene

**Figure 1:**
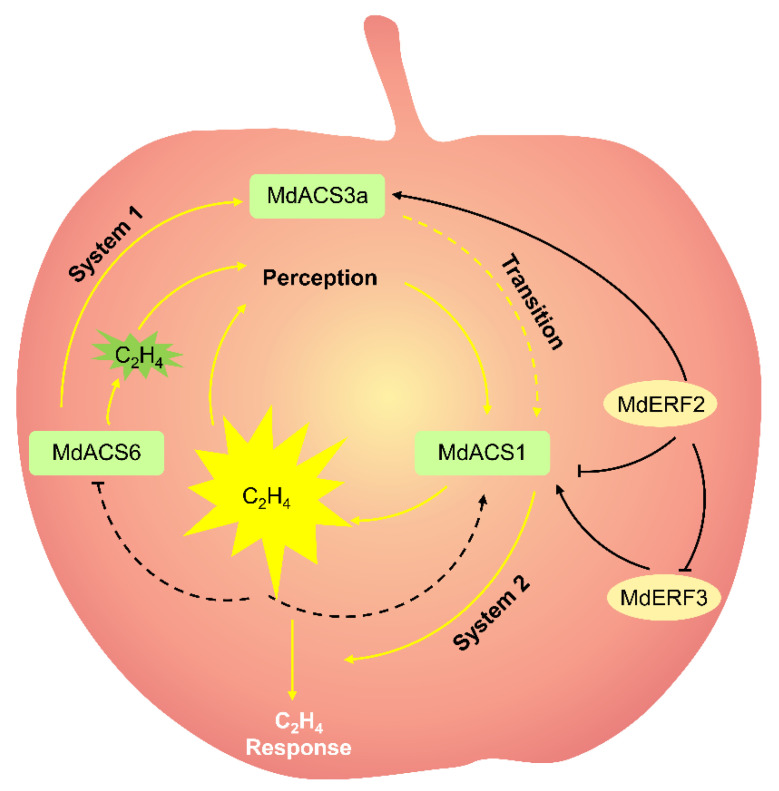
Systems 1 and 2 ethylene in apple fruit ripening. →, promotion; ⊥, suppression; solid arrow, clear regulation mechanism; dotted arrow, unclear regulation mechanism; SAM, *S*-adenosyl methionine; ACC, 1-aminocyclopropane-1-carboxylic acid; C_2_H_4_; ethylene.

Ethylene, a gaseous phytohormone, plays a central role in climacteric fruit ripening. In the apple ripening process, ethylene production gradually increases to a peak, and then gradually decreases; the fruit then moves into the aging stage [3]. The ethylene produced in climacteric fruit is divided into systems 1 and 2. System 1 is mainly responsible for ethylene biosynthesis in young fruit. System 1 ethylene is autoinhibited. System 2 is mainly responsible for ethylene biosynthesis in ripe fruit, and active when climacteric ethylene must be produced. System 2 ethylene in vivo can be autocatalytic by ethylene (Figure 1) [9].

The ethylene is regulated by two pathways in fruit: the biosynthesis pathway and signal-transduction pathway. The ethylene biosynthesis consists of two critical steps: the conversion of *S*-adenosylmethionine (SAM) into 1-aminocyclopropane1-carboxylic acid (ACC) by ACC synthase (ACS), and then the formation of ethylene from ACC by ACC oxidase (ACO) [10]. ACC acts as a direct precursor of ethylene biosynthesis, and its concentrations are closely related to ethylene production [11,12]. Therefore, ACS and ACO are the rate-limiting enzyme in ethylene biosynthesis [3]. Ethylene biosynthesis in plants, especially in fruit, is a complex reaction involving the cooperative action of multiple *ACS* and *ACO* genes. In other words, ethylene biosynthesis in a fruit requires distinct *ACS* genes acting at different developmental processes. In apples, *MdACS6*, *MdACS3a,* and *MdACS1* are three important coordinated *MdACSs* that regulate ethylene biosynthesis during apple-fruit development and maturation [3,13,14]. Of these, *MdACS6* is expressed in the preliminary stage of apple-fruit development, before *MdACS3a* and *MdACS1* are expressed [13], implying that MdACS6 mainly catalyzes the ethylene production in the preliminary stage of apple-fruit development. *MdACS6* expression decreases around 30 days prior to maturation, when *MdACS3a* expression is initiated [14,15]. This suggests that MdACS3a may replace MdACS6 catalysis to meet the demand for ethylene production in apple fruit just prior to maturation. The overexpression of *MdACS6* induces *MdACS3a* expression, indicating that MdACS6 can regulate *MdACS3a* expression [13]. At the onset of ripening, *MdACS1* is abundantly expressed and is responsible for ripening-related ethylene biosynthesis in apple fruit (Figure 1) [2,16]. Considering gene structure, MdACS1 contains an RLSF motif and a C-terminal tail, which is indispensable for mitogen-activated protein kinase 6 and calcium-dependent protein kinase phosphorylation, respectively; however, MdACS6 and MdACS3a have neither of them [2,16]. This may be one of the reasons why in apple fruit, *MdACS6* and *MdACS3a* are expressed during the development stage, while *MdACS1* is specifically expressed during the ripening stage. During apple-fruit ripening, ethylene production is strictly related to *MdACS1* expression [2,17]. Moreover, the differential expression of *MdACS1* allelic forms (*MdACS1-1* and *-2*) among different apple cultivars causes differences in the ethylene production of fruit [17]. All cultivars that were homozygous for *MdACS1-2* produced apples with good long-term storage properties and/or less ethylene production than other *MdACS1* allelic cultivars at the climacteric stage, such as cv. Fuji [2]. Additionally, they have a lower preharvest drop rate than that of other *MdACS1* allelic cultivars by calculating the preharvest fruit drop rate of 40 commercial cultivars [18]. The importance of *MdACS1* in fruit ripening was shown in transgenic apples, in which *MdACS1* was silenced with RNA interference [19]. The silencing of *MdACS1* in apples produced 94% less ethylene during respiration and were significantly firmer than the controls, displaying a longer shelf life [19]. Previous studies have shown that ACO also plays an important role as another rate-limiting enzyme in the ethylene biosynthesis pathway. Silencing *MdACO1* in apples significantly inhibits ethylene production and fruit softening [19]. These findings confirm that the expression of *MdACS* and *MdACO* genes is required for apple-fruit ripening.

In the ethylene signaling transduction pathway, ethylene firstly binds to receptors [20]. Three ethylene receptor families: ethylene resistant (ETR), ethylene response sensor (ERS), and ethylene insensitive 4 (EIN4), have been identified. Nine ethylene receptors (MdETR1, MdETR1b, MdETR2, MdETR5, MdETR101, MdETR102, MdETR105, MdERS1, and MdERS2) were identified in apple [21]. All these receptor genes are expressed in the apple fruit except *MdETR101.* The transcriptional level of *MdETR2*, *MdETR5*, *MdETR102*, *MdERS1,* and *MdERS2* is remarkably induced by exogenous ethylene treatment during apple-fruit ripening [21], suggesting that they are related to fruit ripening. Then, constitutive triple response 1 (CTR1) acts downstream of the receptors [22]. When ethylene does not exist, receptors activate the kinase activity of CTR1 and the downstream progression of signaling is suppressed. When ethylene exists, the receptors no longer activate CTR1, and downstream positive responses, such as ethylene insensitive 2 (EIN2) and the EIN3-like (EIN3/EIL) family, are activated [23]. The EIN3/EIL family is the primary or core transcription factor (TF) binding to the primary ethylene response element (PERE) or EIL conserved binding sequence (ECBS) motif of gene promoters to regulate their transcription [24]. During apple ripening, fruit coloration is accompanied by ethylene biosynthesis [25]. The mechanism is that ethylene treatment evidently promoted fruit coloration as well as *MdEIL1* expression. Ethylene-activated MdEIL1 binds directly to the *MdMYB1* promoter to promote *MdMYB1* expression, anthocyanin accumulation, and fruit coloration [25]. Downstream of EIN3/EIL, the ethylene response factor (ERF) is the secondary TF to trigger ethylene progression. Previous studies identified 259 sequences containing at least one ERF domain in the apple genome [26]. However, ripening-involved *MdERFs* need to be further isolated by expression profiling analyses and gene function identification. For example, MdERF1 and MdERF2 were isolated from ripening apple fruit [27]. *MdERF1* was predominantly expressed in ripening fruit, although a small degree of expression was also detected in non-fruit tissue, while *MdERF2* was specifically expressed in ripening fruit [27]. In apples, the silencing of *MdERF2* led to rapid fruit ripening, while *MdERF2* overexpression led to delayed fruit ripening compared with in the controls [3], indicating that MdERF2 functions as a negative regulator in apple ripening. ERF can exclusively bind to the dehydration-responsive element (DRE) motif or GCC box of promoters of ethylene-responsive genes [3,28]. In apples, as a negative regulator, MdERF2 binds to the DRE motif of the *MdACS1* promoter and suppresses its expression [3]. MdERF3 also binds to the DRE motif of the *MdACS1* promoter but enhances its expression, acting as a positive regulator of apple ripening [3]. MdERF2 functions upstream of MdERF3, where MdERF2 binds to the *MdERF3* promoter and suppresses MdERF3 transcription (Figure 1) [3]. The existence of ERF with negative regulation might balance the speed of fruit ripening, which prevents the fruit from ripening too quickly, and is helpful for attracting animals to disperse seeds. In addition, MdERF4 binds to the *MdERF3* promoter and represses its expression [29]. A mutation (C–G) was identified in the ethylene response factor-associated amphiphilic repression (EAR) motif of the MdERF4 coding region. The EAR mutation of MdERF4 leads to reduced inhibition of *MdERF3* expression, which in turn promotes ethylene biosynthesis [29].

## 3. Auxins

Auxins have been widely studied as growth and development regulators in fruit [30,31]. An increasing number of studies show that auxin also acts as a fruit-ripening regulator. In general, the most abundant free auxin, indole-3-acetic acid (IAA), is seen as the main regulator in fruit [32]. In apple fruit, endogenous IAA contents are extremely high during the initial growth developmental stages, after which IAA contents tend to decline to low levels at the onset of fruit ripening [5].

Signal transduction by auxin is understood well. In the absence of auxin, auxin/indole-acetic acid proteins (Aux/IAAs) interact with auxin response factor (ARF) and suppress their activity, which prevents the downstream progression of signaling. In the perception of auxin, its receptor, transport inhibitor response 1 (TIR1), recognizes auxin, which promotes the interaction between Aux/IAAs and TIR1. Then, Aux/IAAs are removed by ubiquitin-mediated action, so that ARF is released to regulate the expression of downstream genes [33,34]. ARF is described as the key TF in the auxin signaling pathway. In apples, *MdARF5* silencing led to delayed ethylene production and slower ripening, while the overexpression of *MdARF5* substantially promoted ethylene production and ripening compared with controls, indicating that MARF5 functions as a positive regulator of apple ripening [5].

Exogenous synthetic auxin naphthaleneacetic acid (NAA) treatment evidently promoted ethylene production and apple ripening [5]. Auxins both induced ethylene production of apples after commercial harvest (145 DAFB) and promoted ethylene biosynthesis during apple-fruit development. NAA observably induced ethylene production at 115 DAFB, when fruits were not ripe [5]. Previous studies reported that 1-MCP dramatically inhibits apple-fruit ripening [35]. However, 1-MCP followed by NAA treatment restored the ethylene biosynthesis of apple fruit compared with 1-MCP treatment alone [5]. These results indicate that auxins can induce ethylene biosynthesis even when the apple fruit does not have the competence to ripen. The mechanism is that NAA treatment promotes the expression of the ethylene biosynthesis genes *MdACS3a*, *MdACS1,* and *MdACO1,* and the auxin signaling gene *MdARF5.* MdARF5 as a TF binds to the promoters of *MdACS3a*, *MdACS1,* and *MdACO1*, and promotes their expression and ethylene production of apples [5].

The effect of auxins on ethylene biosynthesis can be in a dose-dependent manner in different species. For example, NAA application promoted ethylene biosynthesis and ripening in apples, peaches (*Prunus persica*), and plums (*Prunus salicina*) [5,36,37]. However, auxin treatment suppressed ethylene biosynthesis and delayed ripening in tomatoes (*Solanum lycopersicum*) [38]. Treatment with 100 μM IAA delayed bananas’ (*Musa acuminate*) ripening [39], whereas 57.1 μM IAA had the opposite effect [40].

## 4. Jasmonates 

Most studies on jasmonates (JAs) in plants focused on plants’ response to biotic and abiotic stresses [41]. JAs are also important in fruit ripening [4,42,43]. Kondo et al. [44] reported that the JA concentration was high in the early fruit development of apples, decreased along with fruit growth, and then increased again immediately before maturation. The treatment of methyl jasmonate (MeJA) on apples resulted in increased ethylene production and earlier fruit ripening [4,45,46]. 

JA signal transduction is well-documented [47,48,49], in which the TF MYC is considered to be the main regulator [47]. After silencing *MdMYC2* in apple fruit, ethylene production was evidently lower compared with in the control fruits, and MeJA treatment no longer promoted ethylene [4]. These results suggest that JA-induced ethylene production in apples is regulated by MdMYC2. The mechanism is that JA-activated MdMYC2 directly binds to the promoters of both *MdACS1* and *MdACO1,* and promotes their expression. Additionally, MdMYC2 binds to the *MdERF3* promoter, indirectly activating *MdACS1* transcription. In addition, MdMYC2 interacts with MdERF2 and prevents it from suppressing both *MdERF3* and *MdACS1* [4]. JAs both regulate fruit ripening and affect fruit color development. The exogenous application of JAs can availably enhance color development in apples, but it might shorten the fruit storage period by promoting ethylene production [4,50]. Liu et al. discovered that applying MeJA to apples (cv. Hanfu) 3 weeks before commercial harvest enhanced fruit coloration without affecting fruit firmness and ethylene production during storage [51]. These findings are of great significance to regulate the color and ripening of apple fruit.

## 5. Brassinosteroids 

Brassinosteroids (BRs) are important steroid hormones that promote plant growth and development, for example, cell proliferation, pollen development, fruit ripening, and senescence [52]. The latest study showed that endogenous BRs, including typhasterol (TY), 6-deoxocastasterone (6-deoxoCS), and castasterone (CS), reduced gradually during pear (*Pyrus ussuriensis*) fruit development [6]. The progressive reduction of endogenous BRs during fruit development indicated that BRs might be an inhibitory factor of fruit ripening. The application of 3 μM epibrassinolide (EBR) observably suppressed ethylene production and effectively maintained fruit firmness during the apple-fruit storage period. In contrast, a treatment of 10 μM brassinazole (Brz), an inhibitor of BR biosynthesis, evidently promoted ethylene production and decreased fruit firmness in apples [6]. In pears, exogenous EBR and Brz treatment has the same effect as in apples [6]. These findings suggest that the mechanism by which BR suppresses ethylene biosynthesis in apples and pears may be conserved.

The BR signaling pathway in plants has been well-studied. Following biosynthesis, BR is accepted by the receptor brassinosteroid insensitive 1 (BRI1). BRI1 interacts with and transphosphorylates BRI1-associated kinase 1, which allows for BRI1 to phosphorylate BR signaling kinase 1 (BSK1). Next, the BRI suppressor 1 (BSU1) is activated by the phosphorylated BSK1, and then dephosphorylates and inhibits brassinosteroid insensitive 2 (BIN2), resulting in unphosphorylated brassinazole-resistant 1 (BZR1) and its homolog, BRI1-EMS suppressor 1 (BES1), moving to the nucleus [53]. BZR1/BES1 is an important TF downstream to the BR signal-transduction pathway, which regulates the expression of BR-responsive genes by binding their promoters. In apples and pears, the exogenous treatment of BR activated *BZR1* expression [6]. Silencing *PuBZR1* in pears significantly promoted ethylene production, and EBR no longer suppressed ethylene biosynthesis compared to that in the control fruit [6]. The action of ethylene biosynthetic genes was higher in silencing *PuBZR1* fruit compared with in the control fruit [6]. This finding indicates that PuBZR1 action is vital for BR-suppressed ethylene production in pears. Further research shows that a high concentration of BRs activates the expression of *PuBZR1*, PuBZR1 suppresses the enzyme activity of PuACO1 by directly interacting with it in the cytoplasm, and the expression of *PuACO1* and *PuACS1a* in the nucleus by binding their promoters, thereby suppressing ethylene production and fruit ripening. Moreover, PuBZR1 indirectly suppresses the expression of *PuACO1* and *PuACS1a* through its suppressed action on PuERF2. These results suggest that BR suppresses ethylene production and fruit ripening by BR-activated BZR1 suppressing ACO1 activity and the expression of *ACO1* and *ACS1*.

## 6. Abscisic Acid 

Abscisic acid (ABA) has long been described to be primarily involved in the ripening process of nonclimacteric fruit [54,55]. In recent years, an increasing number of studies discovered that ABA also regulates the ripening of climacteric fruit [56,57,58]. The endogenous ABA concentration is low in green fruit but increases during apple-fruit ripening [59,60]. The ABA concentration of apple fruit (cv. Red Winesap) is maintained at a range of 100–120 ng g^−1^ FW in 60–130 DAFB. Afterwards, apple fruit show a sharp increase in the ABA concentration to a level of 230–240 ng g^−1^ FW, and this reaches the maximal observed levels of about 300 ng g^−1^ FW just before commercial harvest (172 DAFB) [59]. Studies showed that the maximal endogenous ABA precedes a burst of ethylene in apple fruit [59]. These results indicated ABA may be the other regulatory factor upstream of ethylene for apple-fruit ripening.

So far, there is not much information about the mechanisms through which ABA regulates apple-fruit ripening. In Hongyu apples, three different stages of fruit that were harvested at 120 DAFB considered to be commercially ripened were identified: 110 DAFB as preripening and 120 DAFB followed by five days storage at 20 °C as postripening samples were used for RNA-Seq analysis. Differentially expressed gene-analysis results showed that, in the ABA biosynthesis pathway, the expression of key catabolizing gene encoding *9-cis-epoxycarotenoid dioxygenase (NCED1*) was higher in postripening apples, suggesting that ABA might play a regulatory role in apple ripening [7]. Moreover, another gene encoding protein phosphatase 2C and involved in ABA signaling showed higher expression in postripening apples, and several genes encoding serine or threonine protein kinases showed lower expression [7]. These findings suggest that ABA may mediate protein-phosphorylation modification to affect apple-fruit ripening. However, the specific mechanism by which ABA affects apple-fruit ripening is unclear. 

## 7. Gibberellins 

Gibberellins (GAs) are a category of tetracyclic diterpenoid hormones in higher plants regulating a wide range of developmental processes [61,62,63,64,65]. Recent studies on GAs mainly focused on seed development, flowering, and fruit set and development because of the high concentration of GAs found in flowers and immature fruit [42,54]. Among several hundred plant GAs, only a limited number are bioactive in higher plants, such as GA_1_, GA_3_, GA_4,_ and GA_7_. GA_1_ and GA_4_ are highly abundant, whereas GA_3_ and GA_7_ are less abundant [64].

In fruit, GAs accumulate during early fruit development but decrease to a low concentration during fruit ripening [42,64]. Injecting the GA biosynthesis inhibitor prohexadione-Ca into mature green tomatoes accelerated fruit ripening [64]. Additionally, exogenous GA_3_ treatment can reduce ethylene production and depress the ripening of various climacteric fruit, such as bananas, persimmon (*Diospyros kaki*), mangos (*Mangifera indica*), and tomatoes [40,64,66,67,68]. In GA_3_-treated tomatoes, the transcriptional levels of *SlACS2*, *SlACS4,* and *SlACO1* were depressed, and *SlETR3*, *SlETR4*, and *SlEIN2* expression showed a dramatic reduction, indicating that GAs inhibit ethylene biosynthesis and perception during fruit ripening [64]. These results demonstrate that GAs are an inhibitor of fruit ripening. However, the regulation of GAs in apple-fruit ripening has rarely been studied. The ethylene production of apple fruit was remarkably suppressed by 200 μM GA3. We discovered an AP2 family gene, *MdRAV1,* activated by GA3 treatment in apple fruit. Silencing *MdRAV1* in apple fruit led to rapid fruit ripening compared with in the controls, followed by GA3 treatment, and fruit ethylene production was no longer suppressed (unpublished data). In apples, the inactivation of GAs was controlled by a gene encoding *gibberellin 2-beta-dioxygenase* (*GA2ox1*) observed to be high in postripening apples that were harvested at 120 DAFB followed by five days of storage at 20 °C. Knowledge on the mechanisms regarding how GAs regulate apple-fruit ripening remains limited.

## 8. Conclusions and Perspectives

Due to the shorter ripening period, apples are harvested at the commercial maturity stage for a longer shelf life and proper marketing supply. The transition from growth to maturation of fruit is characterized by alterations in the phytohormonal profiles to drastically terminate fruit expansion and promote fruit ripening [69]. A clear understanding of these phytohormonal shifts in apples is meaningful and crucial for regulating the period from commercial to physiological ripening. Moreover, phytohormonal regulation in apple ripening is of great significance for regulating the market supply in the off-season of fruit production.

Fruit ripening is a complicated physiology and biochemistry reaction involving well-organized regulation by multiple hormones, and accompanied by subtle changes of metabolic and physiological traits [70]. Ethylene is specifically required for the ripening of climacteric fruit [3]. The biosynthesis of ethylene in climacteric fruit is divided into systems 1 and 2 [9]. However, the mechanism for system 1 ethylene shifting to system 2 ethylene is not clear (Figure 1). Understanding this mechanism is a major focus of research on fruit ripening.

**Figure 2:**
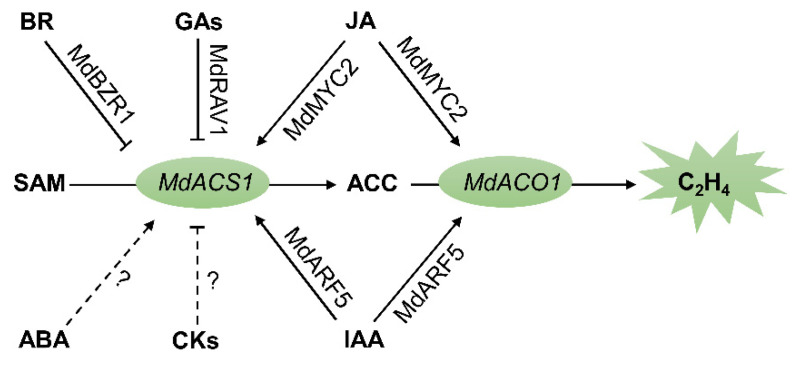
Phytohormone involved in regulating apple fruit ripening. →, promotion; ⊥, suppression; solid arrow, clear regulation mechanism; dotted arrow, unclear regulation mechanism; SAM, *S*-adenosyl methionine; ACC, 1-aminocyclopropane-1-carboxylic acid; C_2_H_4_; ethylene.

Current information indicates that ethylene could be the destination of hormonal crosstalk during apple-fruit ripening. Ethylene signaling in apple-fruit ripening is tightly coordinated under the influence of multiple phytohormones [71]. Cytokinins (CKs) have crucial functions in various phases of plant growth and development as a major phenomenon [72], but studies on the effects of CKs on apple fruit ripening are limited. Other plant hormones primarily act through minor adjustments to ethylene’s action during apple-fruit ripening (Figure 2) [7]. However, available information is limited about the crosstalk of multihormones during apple-fruit ripening. Given the complexity of apple-fruit ripening processes, exploring the basic molecular mechanisms of their regulation by crosstalk among hormones is more difficult. More work is required to elucidate the molecular basis of multihormonal crosstalk, and this is becoming a major focus of research on fruit ripening.
